# Integrative multi-omics and machine learning identify CHRNA1 putative circadian-immune hub in COPD

**DOI:** 10.1371/journal.pone.0353838

**Published:** 2026-07-31

**Authors:** Lan Zhang, Zhifei Li, Tiansheng Xia, Tingting Yang, Jia Fu, Yan Lu, Jiayi Xu, Kaiyu Han

**Affiliations:** Department of Respiratory and Critical Medicine, The Second Affiliated Hospital of Harbin Medical University, Harbin, China; University of Minnesota Medical School, UNITED STATES OF AMERICA

## Abstract

**Background:**

Circadian rhythm disruption is increasingly recognized as a contributor to chronic inflammatory disorders; however, its specific significance and underlying mechanisms in chronic obstructive pulmonary disease (COPD) remain unclear. This study aimed to identify circadian rhythm-associated biomarkers in COPD and explore their diagnostic value, immune correlations, and therapeutic potential.

**Methods:**

This study integrated four lung transcriptomic datasets from the public GEO database (GSE151052, GSE38974, and GSE76925 as the discovery set, and GSE47460 as the validation set). Differentially expressed circadian rhythm‑related genes (DECRRGs) were identified by intersecting differentially expressed genes with circadian rhythm‑related genes. Functional enrichment analyses (GO and KEGG) were performed, and three machine learning algorithms were applied to screen for signature DECRRGs. An exploratory risk stratification model based on multivariate logistic regression was constructed and evaluated. Immune cell infiltration was assessed using CIBERSORT, and single-cell RNA sequencing analysis was conducted to localize the distribution of key circadian rhythm genes within specific lung cell populations. Finally, the expression of a core gene CHRNA1 was validated by qRT-PCR in peripheral blood samples from COPD patients and healthy controls.

**Results:**

We identified eight circadian rhythm-associated feature genes, among which CHRNA1 emerged as a consistently upregulated hub gene in COPD. An exploratory risk stratification model based on these genes exhibited good discriminatory ability in the discovery cohort (AUC = 0.856, 95% CI: 0.806–0.902). Differential expression of CHRNA1 was validated in an independent cohort and correlated significantly with pro-inflammatory immune infiltration, including increased M1 macrophages and CD8 ⁺ T cells. Single-cell transcriptomics further localized CHRNA1 expression predominantly within B cells in COPD lung tissue. In silico drug screening and ceRNA network analysis predicted potential therapeutics (e.g., amitriptyline, rocuronium bromide) and regulatory miRNAs/lncRNAs. Finally, qRT-PCR confirmed a marked upregulation of CHRNA1 in peripheral blood from COPD patients (*p* < 0.0001).

**Conclusions:**

Our findings suggest that CHRNA1 may serve as a candidate circadian rhythm‑associated immunomodulator in COPD. It demonstrates consistent upregulation across cohorts and shows a significant association with pro‑inflammatory immune infiltration. Single-cell analysis revealed that CHRNA1 is predominantly expressed in pulmonary B cells. The exploratory risk stratification model and predicted therapeutic candidates highlight the translational potential of targeting circadian disruption in COPD, though prospective validation is needed before clinical application.

## 1. Introduction

Chronic obstructive pulmonary disease (COPD) is a prevalent respiratory disorder characterized by persistent airflow limitation and symptoms including dyspnoea, cough, and sputum production [1]. The Global Burden of Disease (GBD) Study reported a rising worldwide prevalence from 100.54 million cases in 1990 to 213.39 million in 2021, with disability-adjusted life-years (DALYs) increasing from 56.86 million to 79.78 million over the same period [2]. COPD diagnoses among people aged 25 and older are predicted to increase by 23% between 2020 and 2050, reaching 600 million cases by the middle of the century [3]. In China, driven by population aging and ambient air pollution, the prevalence has steadily risen, reaching 8.6% and 13.7% among individuals aged 20 years and 40 years, respectively [4,5]. This escalating disease burden imposes substantial socioeconomic costs, yet current diagnostic and therapeutic paradigms remain inadequate.

COPD pathogenesis reflects complex gene–environment interactions dominated by tobacco smoke. Despite the Global Initiative for Chronic Obstructive Lung Disease (GOLD 2025) advocating combined interventions—combining pharmacological and non-pharmacological interventions (e.g., smoking cessation, pulmonary rehabilitation, and procedural treatments) to alleviate symptoms and reduce exacerbations, clinical outcomes have improved only marginally [6].This therapeutic stagnation underscores the urgent need to explore novel pathogenic pathways. One such overlooked avenue is circadian biology. Notably, COPD manifestations mirror the circadian exacerbation pattern observed in asthma: dyspnoea, cough and sputum production peak in the early morning across all disease stages [7].These symptoms persist throughout all disease stages, significantly impairing patients’ quality of life and health status [8]and are associated with an increased risk of acute exacerbations [9]. Moreover, exacerbations themselves exhibit diurnal clustering, with nocturnal deterioration more frequent in elderly male smokers [10]. This rhythmic pattern suggests a potential role for the circadian clock system in the pathophysiology of COPD. Therefore, treatment and management strategies should account for circadian influences to more effectively control symptoms and prevent acute exacerbations.

The circadian rhythm in mammals is regulated by the central pacemaker located in the suprachiasmatic nucleus (SCN) of the hypothalamus, in coordination with peripheral biological clocks in various tissues. The SCN integrates light signals and coordinates peripheral clocks through neuroendocrine pathways, synchronizing physiological activities with external light-dark cycles [11,12]. Its core molecular mechanism involves transcription-translation feedback loops (TTFLs) formed by clock genes, which precisely regulate the 24-hour rhythm and influence critical processes such as sleep-wake cycles, inflammation, immunity, and metabolism. Biological clock homeostasis is crucial for maintaining health; circadian disruption can impair these functions and increase susceptibility to inflammatory diseases [13].Previous studies indicate that circadian dysfunction contributes to core pathological processes in COPD. It affects oxidative stress, inflammatory responses, metabolism, and lung function, potentially exacerbating disease severity in elderly COPD patients [14]. For instance, reduced SIRT1 destabilises the molecular clock and amplifies inflammatory responses in COPD [15], whereas BMAL1 confers protection against smoke-induced pulmonary inflammation [16]. Recent studies further reveal that circadian rhythm gene expression correlates with enhanced COPD-associated pulmonary inflammatory genes, with core clock genes negatively correlated with pro-inflammatory genes [17].

However, existing studies have significant limitations: most focus on single genes, lacking systematic analysis of circadian gene networks; moreover, they fail to elucidate the spatiotemporal mechanisms of dynamic interactions between circadian oscillations and the immune microenvironment (e.g., macrophage polarization, T cell infiltration). Consequently, the crucial spatiotemporal crosstalk between the circadian transcriptome and the immune microenvironment in COPD remains largely unexplored and represents a critical knowledge gap.

To address this, the present study integrated multi-cohort lung tissue transcriptomic data (n = 300) and employed an ensemble machine learning strategy (LASSO regression, support vector machines, random forests) to identify COPD-associated circadian rhythm genes. Through systematic analysis of the spatiotemporal dynamics of immune infiltration and ceRNA regulatory networks, we identified the nicotinic acetylcholine receptor alpha 1 subunit (CHRNA1) as the focal gene of this study. This work provides a circadian-immunity framework for understanding COPD pathogenesis and highlights CHRNA1 as a candidate target for future mechanistic and therapeutic investigations.

## 2. Methods

### 2.1. Data acquisition and preprocessing

This study utilized four publicly available lung tissue microarray datasets related to chronic obstructive pulmonary disease (COPD) from the Gene Expression Omnibus (GEO) database (accession URL: http://www.ncbi.nlm.nih.gov/geo/): GSE151052 (COPD: n = 77; Control: n = 40), GSE38974 (COPD: n = 23; Control: n = 9), GSE76925 (COPD: n = 111; Control: n = 40), and GSE47460 (COPD: n = 144; Control: n = 91). GSE151052, GSE38974, and GSE76925 formed the discovery cohort; GSE47460 served as the independent validation cohort.

Expression matrices were downloaded using the GEOquery package. Probe IDs were mapped to gene symbols based on platform annotations, and multiple probes per gene were summarized by averaging using the avereps function. After log_2_(expression + 1) transformation, quantile normalization was performed using limma::normalizeBetweenArrays. Common genes across the three discovery datasets were subjected to batch effect correction using ComBat (sva package). To preserve biological differences between COPD and control samples while removing technical batch effects, the model matrix including group information (mod = model.matrix(~group)) was specified in the ComBat function. The validation cohort GSE47460 was processed separately following the same pipeline to avoid data leakage. Principal component analysis (PCA) was performed to confirm the effectiveness of batch correction.

Because GSE151052 contained non‑independent lung samples derived from the same source lungs (10 COPD explanted lungs and 5 control lungs), a sensitivity analysis was conducted using GSE38974 and GSE76925 as the discovery cohort (COPD = 134, control = 49) and GSE151052 as the validation set (COPD = 77, control = 40) to validate the robustness of CHRNA1.

### 2.2. Identification of circadian rhythm-related gene sets

Differential expression analysis between COPD patients and healthy controls in the batch‑corrected discovery cohort was performed using the limma package (v3.58.1). Differentially Expressed Genes (DEGs) were identified using the criteria of |log_2_FC| > 0.585 and a Benjamini‑Hochberg‑adjusted P value (FDR) < 0.05. The overall patterns and statistical significance of gene expression differences were visualized using heatmaps generated with the pheatmap package and volcano plots generated with the ggplot2 package.

To identify circadian rhythm‑related genes, nine circadian gene sets (Gene Set IDs: M22067, M14104, M13729, M12080, M18009, M95, M938, M39605, M36019) were retrieved from MSigDB. After removing duplicate genes, these sets were consolidated into a reference list of 1,010 unique genes (S1 Table). By intersecting the Differentially Expressed Genes (DEGs) with the 1,010 circadian genes, a set of Differentially Expressed Circadian Rhythm-related Genes (DECRRGs) was identified and visualized using a Venn diagram (VennDiagram).

A heatmap of DECRRG expression was generated using pheatmap. The Pearson correlation matrix among DECRRGs was calculated and visualized with corrplot, and statistical significance was assessed using the cor.mtest function.

### 2.3. Functional enrichment analysis

Functional enrichment analysis of DECRRGs was performed using clusterProfiler (v4.10.1). Gene symbols were converted to Entrez IDs via org.Hs.e.g.,db (v3.18.0). Gene Ontology (GO) enrichment analysis was conducted using the enrichGO function in clusterProfiler with the human annotation database (OrgDb = “org.Hs.e.g.,db”), covering the three ontology categories: Biological Process (BP), Molecular Function (MF), and Cellular Component (CC). Kyoto Encyclopedia of Genes and Genomes (KEGG) pathway enrichment analysis was performed using the enrichKEGG function. An unadjusted P value < 0.05 and an FDR < 0.05 were set as significance thresholds. The results were visualized as bar plots, bubble plots, and gene‑pathway networks.

### 2.4. Machine-learning feature selection

To identify robust circadian rhythm signature genes associated with COPD, an ensemble feature selection strategy integrating LASSO regression, SVM‑RFE, and random forest (RF) algorithms was employed.

LASSO logistic regression was implemented using the glmnet package (v4.1.8) with family = “binomial” and alpha = 1. The optimal penalty parameter λ was determined via 10‑fold cross‑validation, and genes with non‑zero coefficients at λ_min were retained. The SVM‑RFE algorithm was implemented using the e1071 package (v1.7.16), and feature importance was evaluated using 10‑fold cross‑validation. RF analysis was performed using the randomForest package (v4.7.1.2), with an initial forest of 500 trees and mtry set to the default value. Gene importance was assessed by the mean decrease in Gini index, and genes with scores > 10 were retained. Consensus genes identified by all three methods were designated as core feature genes.

### 2.5. ROC analysis and nomogram construction

A multivariate logistic regression model was constructed based on the eight feature genes using the glm function. The ROC curve was plotted, and the AUC along with its 95% confidence interval was calculated using the pROC package (v1.18.5). A nomogram was developed from the fitted model using the rms package (v6.7.1), and model reliability was assessed by generating calibration curves via 1,000 bootstrap resampling iterations.

### 2.6. Signature-gene visualisation

The expression profiles of the selected genes and the corresponding clinical group information were extracted from the normalized expression matrix. Differential expression boxplots for each feature gene were generated automatically using the ggpubr package. Comparisons between groups were evaluated using the Wilcoxon rank-sum test. Statistical significance was annotated as follows: ***P < 0.001, **P < 0.01, *P < 0.05, ns *P* ≥ 0.05.

### 2.7. Independent-cohort validation

To validate the expression stability of the core signature genes in COPD, an independent validation cohort (GSE47460, comprising 144 COPD patients and 91 healthy controls) was employed. Differential expression analysis between the COPD and control groups was performed using the Wilcoxon rank-sum test, and the results were visualized as boxplots.

### 2.8. Gene-set enrichment analysis (GSEA)

Gene set enrichment analysis (GSEA) for each feature gene was performed using clusterProfiler (v4.10.1).COPD patients were stratified into high‑ and low‑expression groups based on the median expression level of each gene. A pre‑ranked gene list for GSEA was generated by calculating the fold change of expression for each gene and sorting them in descending order. Enrichment analysis was conducted using the MSigDB C2 curated gene set (c2.cp.kegg.Hs.symbols.gmt), with an unadjusted P value < 0.05 set as the significance threshold.

### 2.9. Drug screening and ceRNA network construction

The Drug Signature Database (DSigDB, version 1.0; https://dsigdb.tanlab.org/DSigDBv1.0/) was interrogated to construct a gene–drug regulatory network, which was visualized using Cytoscape.

MiRNAs targeting the feature genes were predicted using the miRanda, miRDB, and TargetScan databases, followed by the prediction of potential interacting lncRNAs. A ceRNA network was then constructed by integrating miRNA–mRNA and lncRNA–miRNA interactions.

### 2.10. Immune-cell infiltration

The CIBERSORT algorithm (v1.03) was applied to estimate the abundances of 22 immune cell subtypes in the integrated dataset (GSE151052, GSE38974, and GSE76925). Differences in immune cell proportions between the control and COPD groups were evaluated using the Wilcoxon rank‑sum test and visualized as boxplots. Spearman rank correlation analysis was performed to assess interactions among immune cell types within the COPD group, and the resulting correlation matrix was visualized as a heatmap. Additionally, Spearman correlation coefficients between the expression level of each key gene and the infiltration level of each immune cell type were calculated.

### 2.11. Single-cell RNA sequencing data processing

A previously published single-cell transcriptomic dataset of idiopathic pulmonary fibrosis, GSE136831 [18], was used in this study. This dataset comprises lung tissues from 32 IPF patients, 18 COPD patients, and 28 healthy controls, encompassing a total of 312,928 cells. For this study, only the COPD and healthy control samples were analyzed; IPF samples were excluded. To investigate the cell type‑specific expression pattern of CHRNA1 in COPD, the analysis focused on the COPD and healthy control samples from this dataset. The expression distribution of CHRNA1 across distinct cell types was visualized using a dotplot.

### 2.12. Experimental validation (RNA extraction and qRT-PCR)

Peripheral blood samples were collected from 19 patients with chronic obstructive pulmonary disease (GOLD stages I–IV) and 19 healthy control volunteers. This study was reviewed and approved by the Ethics Committee of the Second Affiliated Hospital of Harbin Medical University (Approval No.: YJSKY2024−029). All procedures were performed in accordance with the ethical standards of this institutional committee and with the principles of the 1964 Declaration of Helsinki and its later amendments. Written informed consent was obtained from all individual participants (COPD patients and healthy controls) prior to their inclusion in the study.

Total RNA was extracted with TRIzol™ reagent, quantified (NanoDrop 2000; A260/280 > 1.8) and integrity-checked (Agilent 2100; RIN > 7). cDNA was synthesised from 2 µg RNA (PrimeScript™ RT Kit, Takara). CHRNA1 and GAPDH were quantified by SYBR Green qPCR (TB Green™ Premix Ex Taq™ II, Takara) on a QuantStudio 6 Flex system. Primers:

CHRNA1 F 5′-CCGAGGTGAAAAGTGCCATCGA-3′
CHRNA1 R 5′-TCCGAGGAGTATGTGGTCCATC-3′
GAPDH F 5′-GTCTCCTCTGACTTCAACAGCG-3′
GAPDH R 5′-ACCACCCTGTTGCTGTAGCCAA-3′

Reactions (20 µL) contained 7.2 µL of TB Green Premix, 10.0 µL of nuclease‑free water, 0.8 µM of each primer, and 2 µL of cDNA. The qPCR program included an initial holding stage at 50.0 °C for 2 minutes, followed by a denaturation step at 95.0 °C for 10 minutes. The cycling stage consisted of 40 cycles with the following conditions: denaturation at 95.0 °C for 15 seconds, annealing and extension at 60.0 °C for 1 minute. After the cycling stage, a melt curve analysis was performed with a continuous temperature increase from 60.0 °C to 95.0 °C. Triplicate technical replicates were averaged and relative expression calculated by 2^(–ΔΔCt) with GAPDH as reference. Normality was rejected by Shapiro–Wilk (*P < 0.05*); group differences were analysed with Mann–Whitney U tests (GraphPad Prism v10.0) and displayed as box-and-whisker plots (median ± IQR) with exact P values.

### 2.13. Statistical analysis

All analyses were performed in the R software environment (v4.3.3). Continuous variables were expressed as medians with interquartile ranges (IQR), and categorical variables as frequencies (percentages). Between‑group comparisons were conducted using the Wilcoxon rank‑sum test for continuous variables and the χ² test for categorical variables. Correlation analyses were performed using Spearman’s rank correlation coefficient. All tests were two‑sided, and a P value < 0.05 was considered statistically significant.

## 3. Results

### 3.1. Differentially expressed circadian rhythm genes

**Fig 1:**
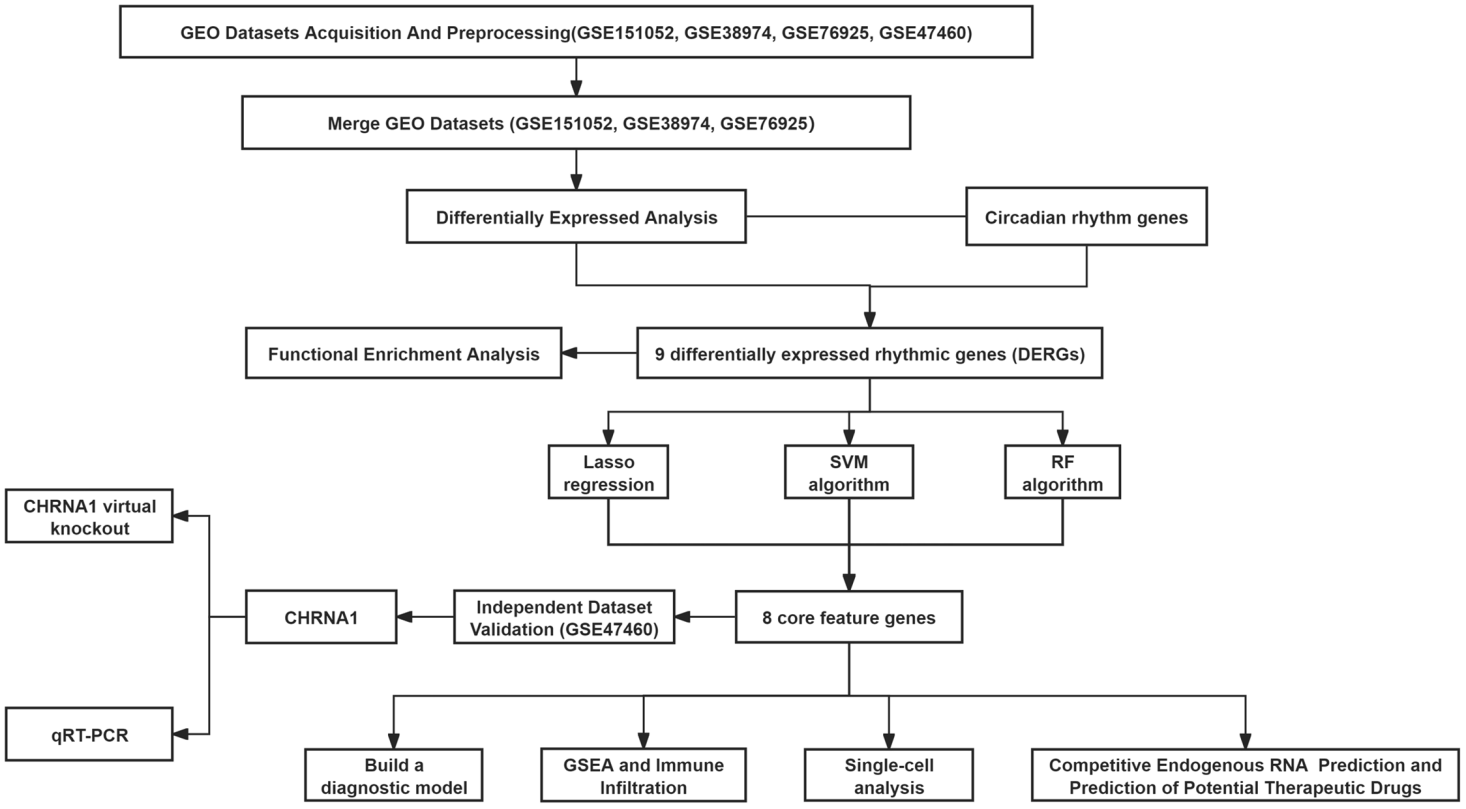
Flowchart of this study.

**Fig 2:**
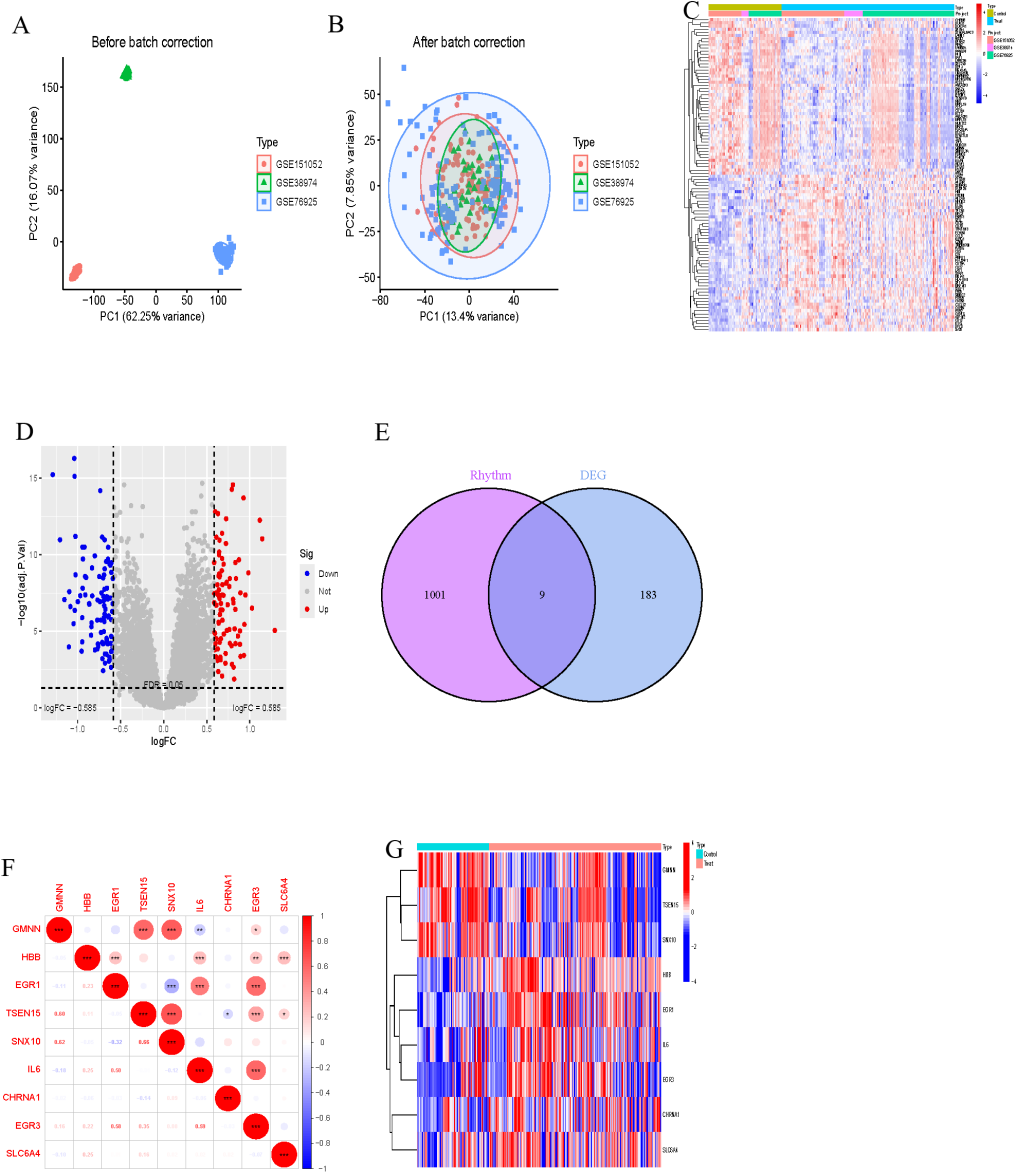
GEO data processing, differential analysis, and visualization of differentially expressed genes. (A) PCA before batch correction. Samples are colored by dataset (GSE151052, GSE38974, GSE76925). (B) PCA after ComBat batch correction, showing improved alignment across datasets. (C) Heatmap of top 50 DEGs. Rows: Z-score normalized genes; columns: samples. Hierarchical clustering applied. (D) Volcano plot of DEGs. Red: upregulated (log_2_FC > 0.585, adj. *P* < 0.05); blue: downregulated (log_2_FC < −0.585, adj. *P* < 0.05); grey: not significant. (E) Venn diagram showing overlap between Circadian-related genes (from MSigDB) (blue) and DEGs (purple). Overlapping set: n = 9 (DECRRGs). (F) Correlation matrix of nine DECRRGs (GMNN, HBB, EGR1, TSEN15, SNX10, IL6, CHRNA1, EGR3, SLC6A4). Red: positive correlation; blue: negative correlation. Numbers represent r values. (G) Expression heatmap of DECRRGs. Top color bar: blue (control), red (COPD). Red: high expression; blue: low expression.

A flowchart of the study is presented in Fig 1. The three discovery datasets (GSE151052, GSE38974, and GSE76925) were integrated to generate a unified expression matrix comprising 300 lung tissue samples (COPD, n = 211; control, n = 89). Principal component analysis (PCA) revealed pronounced platform‑specific separation along PC1 before batch correction (Fig 2A); after ComBat correction, the samples became thoroughly intermingled, confirming that technical disparities had been effectively eliminated (Fig 2B).

Based on the thresholds of |log_2_FC| > 0.585 and FDR < 0.05, a total of 192 DEGs were identified using limma, including 91 upregulated and 101 downregulated genes (Fig 2D). Hierarchical clustering demonstrated that the top 50 DEGs accurately distinguished COPD from control samples (Fig 2C). Intersection of the DEGs with a reference list of 1,010 circadian rhythm‑related genes yielded nine Differentially Expressed Circadian Rhythm Related Genes (DECRRGs): GMNN, HBB, EGR1, TSEN15, SNX10, IL6, CHRNA1, EGR3, and SLC6A4 (Venn diagram, Fig 2E).

Pearson correlation analysis (95% CI) revealed a strong co-expression module comprising GMNN–TSEN15 (r = 0.60) and GMNN–SNX10 (r = 0.62), whereas negative correlations were observed for GMNN–IL6 (r = –0.18) and CHRNA1–TSEN15 (r = –0.14) (Fig 2F). Expression heatmaps (Fig 2G) demonstrated coherent dysregulation of these DECRRGs in COPD.

### 3.2. Functional enrichment of DECRRGs

**Fig 3:**
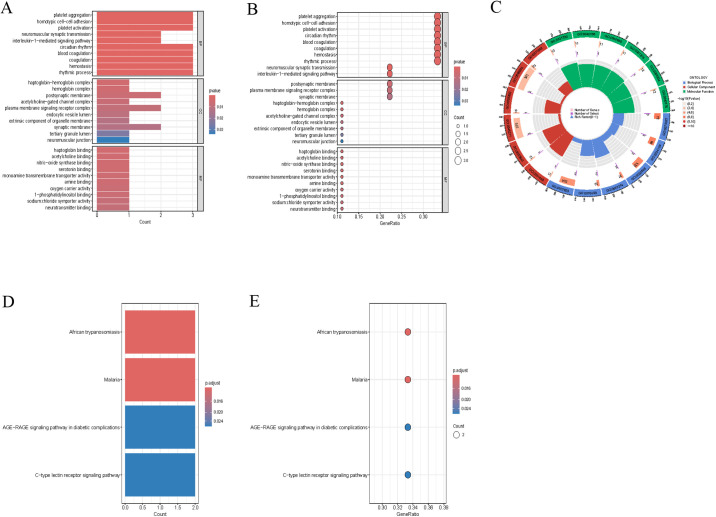
Functional enrichment analysis of gene sets. (A) Bar plot of enriched GO terms (BP, CC, MF). Bar height: gene count; color: adjusted p-value (darker = more significant). (B) Bubble plot of GO enrichment. X-axis: gene ratio; y-axis: -log₁₀(adj. p); bubble size: gene count; color: significance level. (C) Circular visualization of GO enrichment. Outer ring: ontology categories; inner rings: gene counts, enrichment factor, and significance. (D) Bar plot of enriched KEGG pathways. Bar height: gene count; color: adjusted p-value. (E) Bubble plot of KEGG pathway enrichment. X-axis: gene ratio; y-axis: -log₁₀(adj. p); bubble size: gene count; color: significance.

Functional enrichment analysis of the DECRRGs in COPD was performed using Gene Ontology (GO) (Figs 3A-E).

GO enrichment analysis revealed that DECRRGs were primarily involved in circadian rhythm regulation, IL-1 signaling, and neuromuscular transmission. These genes were also associated with ligand-binding activities (e.g., acetylcholine and neurotransmitter binding) and enriched in synaptic structures and membrane receptor complexes (Figs 3A-C).

We further conducted KEGG pathway enrichment analysis, with results displayed in bar and bubble plots (Figs 3D-E). DECRRGs were significantly enriched in two pathways: the C‑type lectin receptor signaling pathway and the AGE‑RAGE signaling pathway in diabetic complications.

### 3.3. Machine-learning–driven feature selection

**Fig 4:**
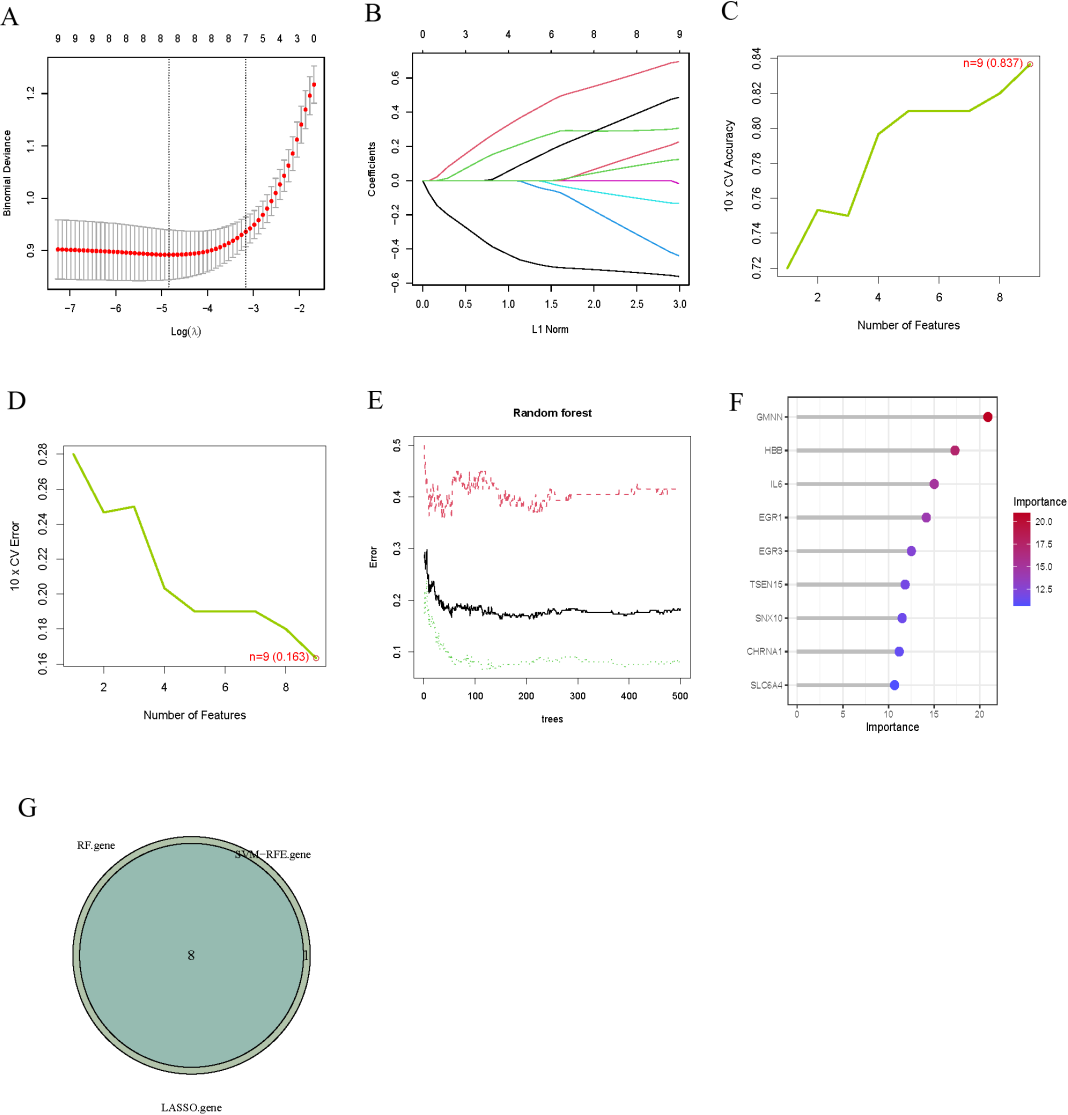
Feature DEGs selection using machine learning algorithms. (A) Lasso cross-validation curve. Vertical line: optimal λ (minimum deviance); shaded area: standard error. (B) Lasso coefficient paths. Vertical line: selected λ; non-zero coefficients retained. (C) SVM-RFE cross-validated accuracy. Optimal feature subset: n = 9 (accuracy 0.837). (D) SVM-RFE cross-validated error. Minimal error (0.163) achieved with 9 features. (E) Random forest error convergence. Error rate stabilizes as number of trees increases. (F) Random forest feature importance. Bubble size and color represent importance score. (G) Venn diagram showing consensus genes identified by Lasso, SVM-RFE, and RF (n = 8).

Using an ensemble machine-learning strategy, we systematically identified COPD-specific circadian rhythm-related genes. Lasso regression screened out 8 key genes (GMNN, HBB, EGR1, TSEN15, SNX10, CHRNA1, EGR3, SLC6A4) (Figs 4A-B), The SVM-RFE algorithm identified nine optimal genes (HBB, TSEN15, CHRNA1, EGR3, GMNN, SLC6A4, EGR1, IL6, SNX10) (Figs 4C-D), and Random Forest identified 9 high-importance genes (GMNN, HBB, IL6, EGR1, EGR3, TSEN15, SNX10, CHRNA1, SLC6A4)(Figs 4E-F). The intersection of the three methods yielded 8 core feature genes (GMNN, HBB, EGR1, TSEN15, SNX10, CHRNA1, EGR3, SLC6A4)(Fig 4G).

Further comparison of gene expression levels between COPD and control groups revealed that HBB, EGR1, CHRNA1, EGR3, and SLC6A4 were significantly upregulated, while GMNN, TSEN15, and SNX10 were markedly downregulated in COPD.

### 3.4. Exploratory risk stratification model construction and validation

**Fig 5:**
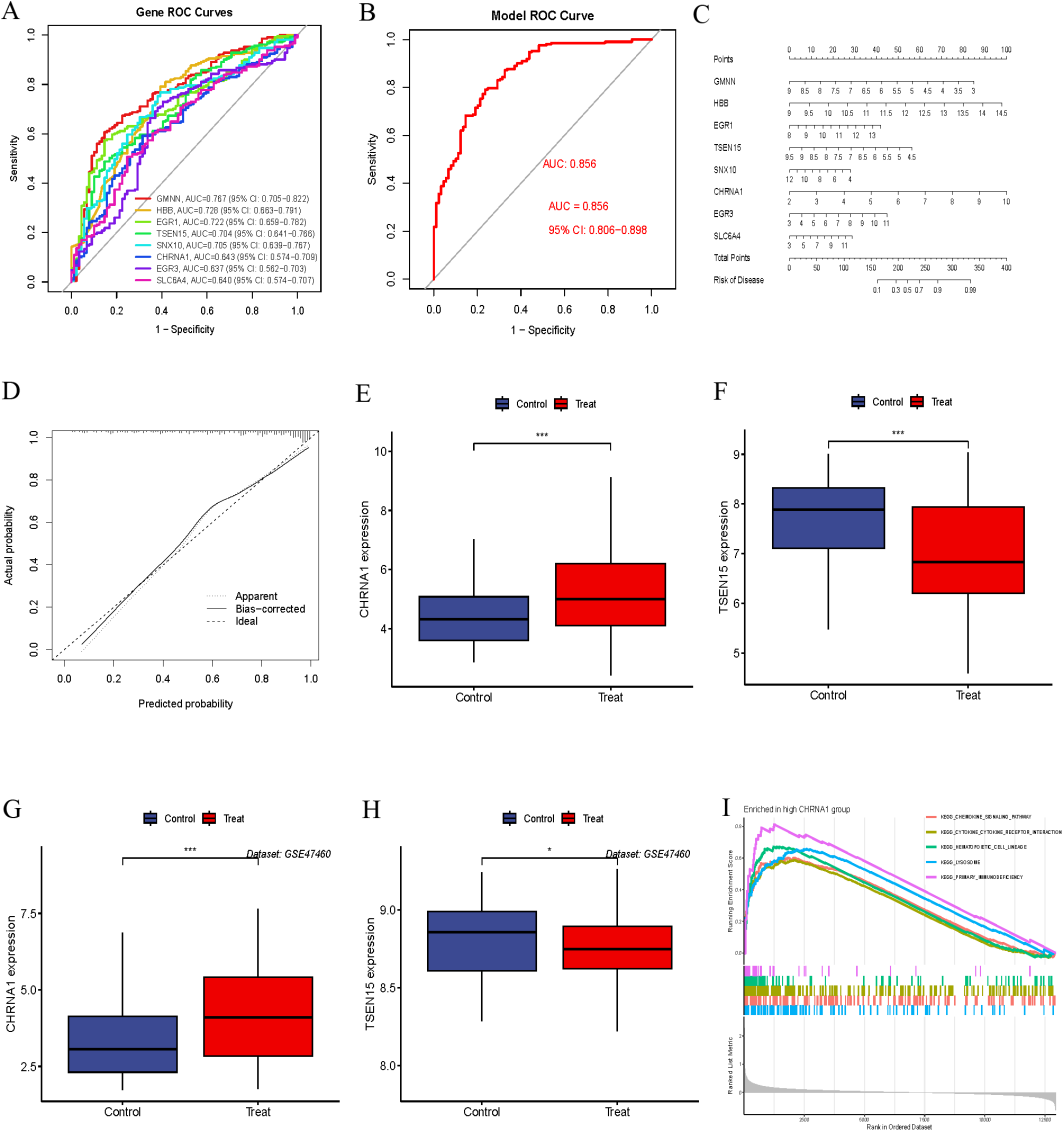
Evaluation of model performance and validation of key gene expression. (A) ROC curves of individual feature genes. AUC values indicate diagnostic performance. (B) ROC curve of the integrated logistic regression model. AUC = 0.856 (95% CI: 0.806-0.902). (C) Nomogram for risk prediction using point-based scoring system. (D) Calibration plot showing agreement between predicted and observed outcomes. (E) CHRNA1 expression in discovery cohort. ***P < 0.001 (Control vs. COPD). (F) TSEN15 expression in discovery cohort. (G) CHRNA1 expression validation in independent cohort (GSE47460). (H) TSEN15 expression validation in GSE47460. (I) GSEA enrichment profile for high CHRNA1 expression group.

Based on eight characteristic circadian rhythm genes (GMNN, HBB, EGR1, TSEN15, SNX10, CHRNA1, EGR3, SLC6A4), a logistic regression-based exploratory risk stratification model was constructed and its performance was preliminarily evaluated using ROC curves. Single-gene analysis revealed that GMNN (AUC = 0.767), HBB (0.728), EGR1 (0.722), TSEN15 (0.704), and SNX10 (0.705) exhibited moderate diagnostic value (AUC > 0.700), while CHRNA1 (0.643), EGR3 (0.637), and SLC6A4 (0.640) demonstrated discriminatory ability (Fig 5A). The combined model demonstrated superior discriminatory performance (AUC = 0.856, 95% CI: 0.806–0.902; Fig 5B).

To explore its potential clinical application value, a multi-gene nomogram was developed (Fig 5C). The calibration curve demonstrated high consistency between the predicted risk and actual observations (Fig 5D).

### 3.5. Independent-cohort validation and sensitivity analysis

In GSE47460, CHRNA1 expression remained significantly elevated in COPD (*P < 0.001*), whereas TSEN15 was markedly reduced (Figs 5E-H). These results were consistent with those observed in the discovery cohort.

**Fig 6:**
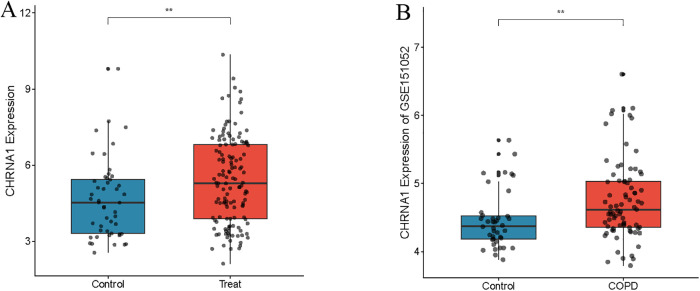
CHRNA1 expression in the discovery and validation cohorts. (A) Box plots of CHRNA1 expression in the COPD and control groups based on the merged dataset (GSE76925 and GSE38974)(**P < 0.01). (B) Box plots of CHRNA1 expression in the COPD and control groups based on the independent validation dataset (GSE151052)(**P < 0.01).

Because GSE151052 contained non‑independent samples derived from the same donor lungs, a sensitivity analysis was conducted using only GSE38974 and GSE76925 as the discovery cohort (COPD = 134, control = 49) and GSE151052 as an independent validation set (COPD = 77, control = 40). CHRNA1 remained significantly upregulated in COPD in both the discovery cohort and the GSE151052 validation set (Figs. 6A-B). These consistent results confirm that the upregulation of CHRNA1 is robust and not attributable to sample non‑independence.

### 3.6. Single-gene enrichment analysis

COPD patients were stratified into high- and low-expression groups based on the median expression level of each feature gene, and single-gene GSEA was performed. The results demonstrated that the gene-centered pathways were primarily involved in immune regulation, metabolic processes, signal transduction, and cellular stress responses. In the CHRNA1 high-expression subset, significant enrichment was observed in pathways including cytokine–cytokine receptor interaction, chemokine signaling pathway, hematopoietic cell lineage, lysosomal function, and primary immunodeficiency (*FDR < 0.05*) (Fig 5I). Conversely, no significantly enriched pathways were detected in the TSEN15-low subgroup.

### 3.7. Therapeutic drug screening and regulatory network

**Fig 7:**
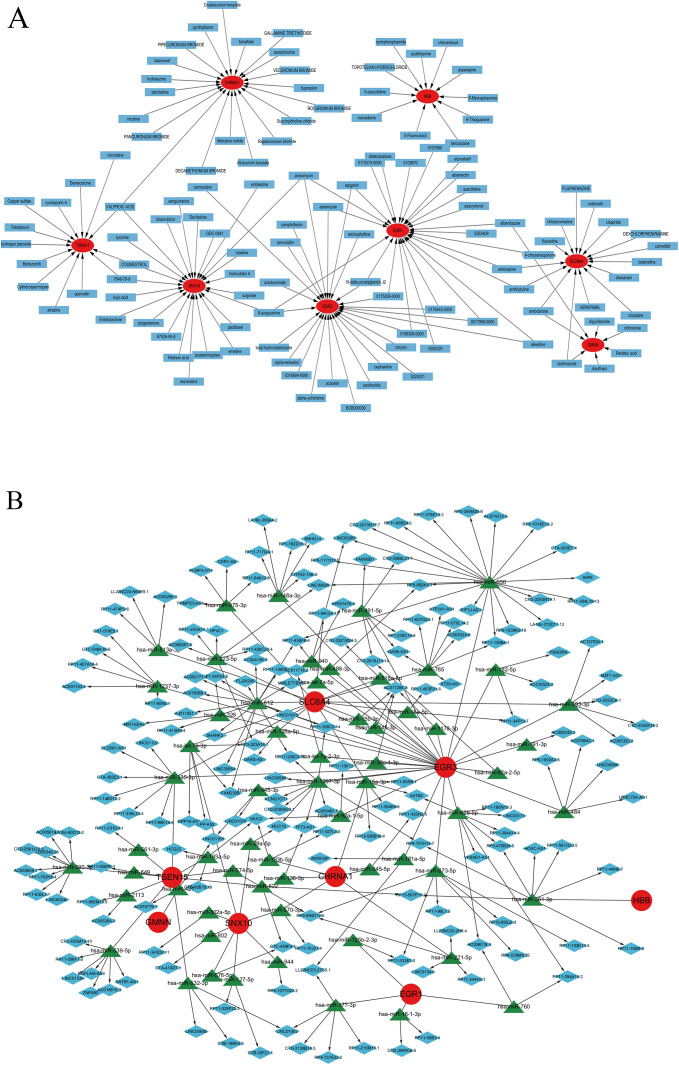
Interaction networks among genes, drugs, and non-coding RNAs. (A) Gene-drug interaction network. Red nodes: candidate genes; blue nodes: pharmacological compounds. Edges represent predicted interactions (e.g., inhibition, activation). (B) ceRNA regulatory network. Red circles: mRNAs; green triangles: miRNAs; blue diamonds: lncRNAs. Edges indicate regulatory relationships (binding or repression).

To explore potential compounds targeting the eight core circadian rhythm genes, the DSigDB database was interrogated and a drug–gene association network was constructed (Fig 7A). In silico predictive analysis revealed potential interactions between several approved pharmaceuticals and multiple circadian rhythm genes, including amitriptyline, fluoxetine, amoxicillin, vecuronium, and rocuronium. These findings are hypothesis‑generating and require functional validation.

To dissect the complex interplay among differentially expressed circadian genes, an integrative competitive endogenous RNA (ceRNA) network was assembled, incorporating predicted interactions among mRNAs, miRNAs, and lncRNAs (Fig 7B). The ceRNA network revealed that the eight feature genes served as key hub nodes, extensively interacting with multiple lncRNAs and miRNAs. Key miRNAs (e.g., hsa‑miR‑193b‑5p and hsa‑miR‑15a‑3p) exhibited broad targeting profiles, while key lncRNAs (TP73‑AS1, RP5‑894P12.5, MUC19) acted as competitive sponges for specific miRNAs, thereby indirectly regulating the expression of the feature genes. This network provides a systemic understanding of molecular circadian disruption in COPD.

### 3.8. Validation of signature gene expression and immune infiltration landscapes

**Fig 8:**
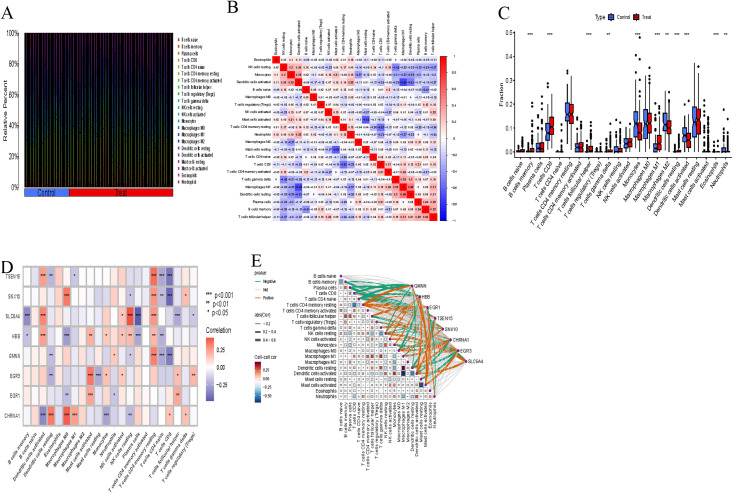
Analysis of immune cell infiltration and its correlation with key genes. (A) Composition of immune cell infiltration. Stacked bar plot shows relative proportions of immune cell types in control (blue) and COPD (red) groups. (B) Correlation among immune cell types. Heatmap of pairwise Spearman correlations in COPD group. (C) Differential immune cell abundance. Box plots comparing immune cell fractions between groups (***P < 0.001, **P < 0.01, *P < 0.05; Mann-Whitney U test). (D) Correlation between gene expression and immune infiltration. Heatmap of Spearman correlations between key genes and immune cells. (E) Regulatory network of genes and immune cells. Rectangles: genes; circles: immune cells. Edges: orange (positive), green (negative), grey (non-significant). Thickness: correlation strength.

CIBERSORT analysis revealed a significant dysregulation of immune cell composition in the lung tissue microenvironment of COPD patients (Figs 8A-C). Specifically, the COPD group exhibited markedly elevated infiltration of M1 macrophages (*P < 0.001*), neutrophils (*P < 0.01*), memory B cells, and CD8 ⁺ T cells (*P < 0.001*) whereas monocyte (*P < 0.001*) and M2 macrophage (*P < 0.01*) infiltration was markedly reduced. Further correlation analysis revealed complex interaction networks among different immune cell types (Fig 8B).

Crucially, we validated significant associations between the expression of these circadian rhythm-related genes and specific immune cell subset infiltration (Figs 8D-E). High CHRNA1 expression positively correlated with infiltration of resting dendritic cells, M0 macrophages, M1 macrophages, CD8 ⁺ T cells, and γδ T cells (*P < 0.001*), while negatively correlating with infiltration of activated dendritic cells and monocytes (*P < 0.001*). In contrast, TSEN15 expression positively correlated with infiltration of resting monocytes and resting CD4 ⁺ memory T cells (*P < 0.001*) and negatively correlated with CD8 ⁺ T cell infiltration (*P < 0.001*).

In summary, altered expression of rhythm‑associated genes in COPD may be associated with the remodeling of specific cellular components in the pulmonary immune microenvironment.

### 3.9. Single‑cell atlas construction and gene localization

**Fig 9:**
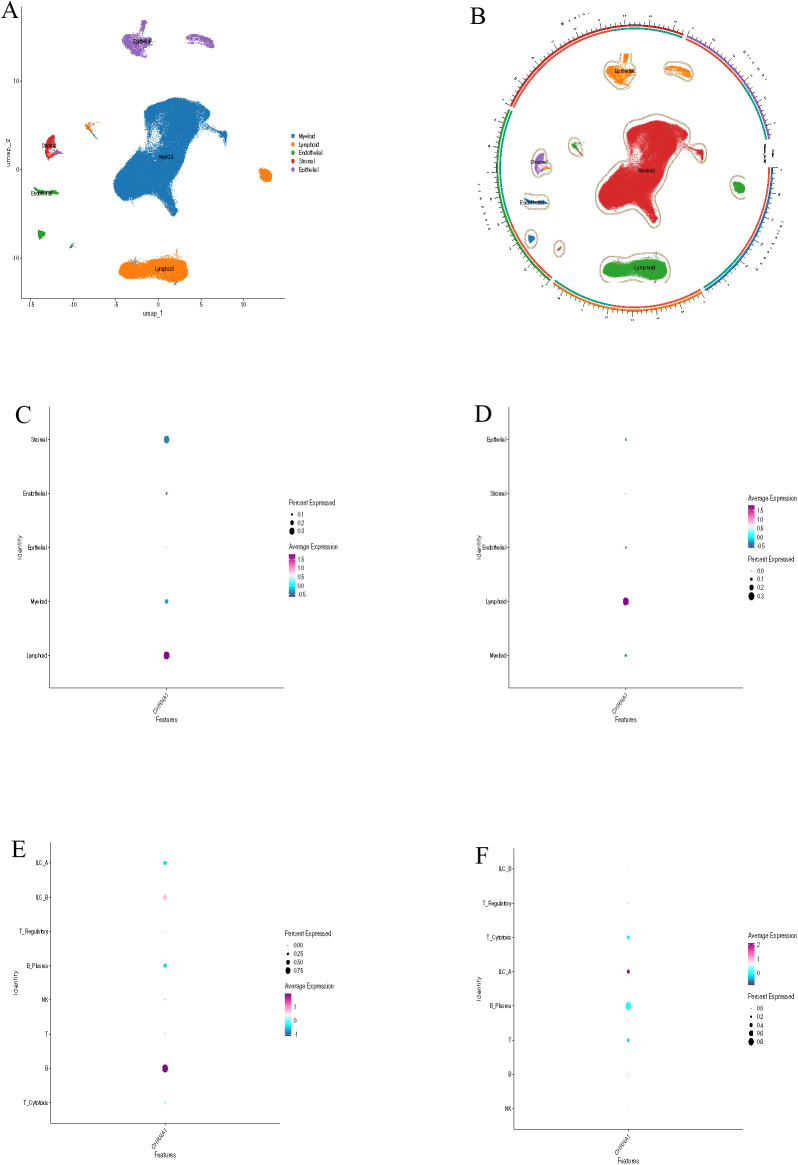
Single-cell level validation of CHRNA1 cellular localization. (A) UMAP plot of lung single cells colored by major cell types (epithelial, endothelial, stromal, myeloid, lymphoid). (B) Circular plot of marker gene expression validating cell type annotation. (C-D) Dot plots of CHRNA1 expression across major cell types in COPD (C) and control (D). Dot size: percentage of expressing cells; color: average expression level. CHRNA1 is predominantly expressed in lymphoid cells. (E-F) Dot plots of CHRNA1 expression in lymphoid subpopulations in COPD (E) and control (F). CHRNA1 shows highest detection frequency and expression level in B cells, particularly in COPD. B: B cells; T: T cells; T_Cytotoxic: cytotoxic T cells; T_Regulatory: regulatory T cells; NK: natural killer cells; ILC: innate lymphoid cells.

To validate the cellular localization of CHRNA1 and its expression changes in COPD at the single‑cell level, we analyzed the GSE136831 dataset, which includes COPD patients and healthy controls. For this analysis, only COPD patients and healthy controls were included. Uniform Manifold Approximation and Projection (UMAP) dimensionality reduction analysis revealed five major cell types: epithelial cells, endothelial cells, stromal cells, myeloid cells, and lymphocytes (Figs 9A-B).

Dotplot analysis demonstrated that CHRNA1 exhibited a higher detection proportion in lymphocytes compared with other cell types (Figs 9C-D). Within lymphocyte subsets, CHRNA1 showed a higher detection frequency in B cells (Figs 9E-F).

### 3.10. Validation of CHRNA1 Expression in Peripheral Blood (qRT‑PCR)

**Fig 10:**
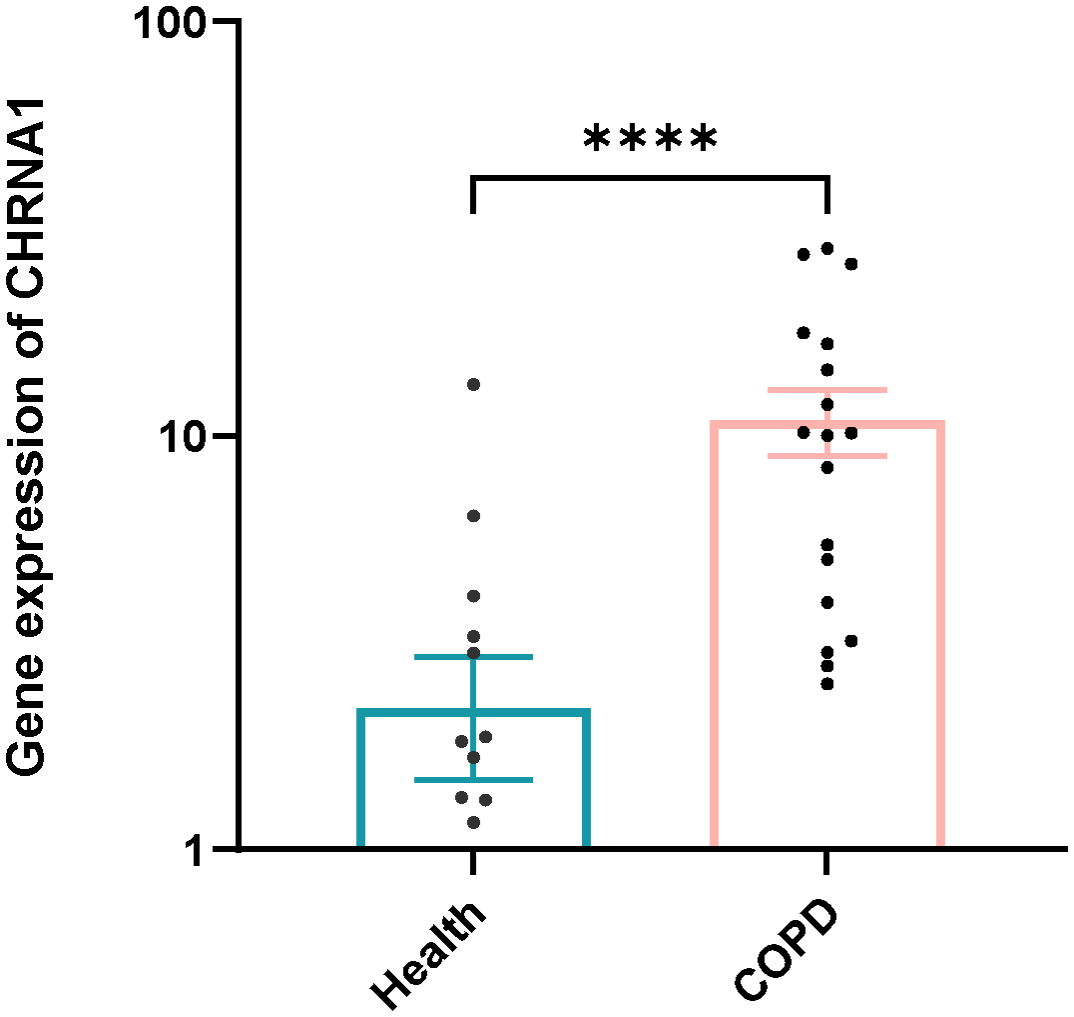
Validation of CHRNA1 expression in clinical samples.

Given the consistent differential expression of CHRNA1 in the independent validation cohort (GSE47460) and its significant correlation with key immune infiltrates, we quantified CHRNA1 transcript levels by qRT-PCR in peripheral blood specimens from 19 COPD patients and 19 healthy controls. CHRNA1 expression was significantly upregulated in peripheral blood samples from COPD patients (n = 19) compared to healthy controls (n = 19) (median relative expression: 10.00 vs. 1.316; Mann-Whitney U = 41, *P* *< 0.0001*) (Fig 10). This experimental validation confirms the marked up-regulation of CHRNA1 in COPD and supports its potential role as a biomarker associated with immune microenvironment modulation.

qRT-PCR confirms significant upregulation of CHRNA1 in peripheral blood from COPD patients (n = 19) compared with healthy controls (n = 19). Data are shown as box plots with individual data points overlaid. The Y‑axis is presented on a log10 scale. **** *P* < 0.0001 (Mann‑Whitney U test).

## 4. Discussion

The core pathological features of COPD are primarily driven by chronic immune-inflammatory processes arising from gene-environment interactions, which are closely associated with abnormal immune cell infiltration and dysregulation [6]. In mammals, circadian rhythms are orchestrated through transcriptional-translational feedback loops centered around core clock genes, which help maintain homeostasis in various physiological processes—including immune and inflammatory responses. Growing evidence suggests that disruption of circadian rhythms can disturb immune regulation and inflammatory balance, thereby increasing susceptibility to a range of diseases [19,20]. In COPD, environmental exposures such as cigarette smoke represent key factors contributing to circadian rhythm disruption. These exposures alter the expression of critical clock genes (e.g., BMAL1, REV-ERBα, PER2, RORα), leading to dysregulation of pulmonary circadian rhythms [14].Such disruption is associated with immune imbalance, oxidative stress, and metabolic dysfunction, thereby directly contributing to the pathogenesis of COPD [21,22]. Although the pivotal role of circadian dysregulation in COPD is recognized, the precise molecular mechanisms by which the circadian system regulates the pulmonary immune microenvironment (particularly immune cell infiltration) remain a core scientific question requiring urgent clarification.

To bridge this gap in understanding the “rhythm-immune interaction” mechanism, this study integrated multi-omics data (including transcriptomics) and applied advanced machine learning strategies. We first identified nine DECRRGs closely associated with COPD. Subsequently, through cross-validation and intersection of three machine learning algorithms (LASSO regression, SVM-RFE, and random forest), we ultimately identified eight circadian rhythm-related genes: GMNN, HBB, EGR1, TSEN15, SNX10, CHRNA1, EGR3, and SLC6A4, all of which are associated with the immune microenvironment. For example, HBB encodes one of the two polypeptide chains responsible for β-hemoglobin subunits. Alterations in this gene may induce sickle cell disease [23], and it also participates in inflammatory responses by influencing the infiltration of immune cells (particularly eosinophils), playing a crucial role in disease onset and progression [24]. Similarly, EGR1 (Early Growth Response 1) is implicated in mediating pulmonary inflammation and contributes to COPD development in smokers [25]. This study selected CHRNA1 for experimental validation because it simultaneously meets the following criteria: (1) highest expression consistency across discovery and validation cohorts (p < 0.001); (2) it demonstrated statistically significant associations in both neuromuscular and immune pathways; (3) it possessed predicted pharmacological targetability (e.g., the nAChR antagonist rocuronium [26]). Previous WGCNA bioinformatic studies have identified CHRNA1 as a COPD hub gene associated with ECM dysregulation [27]. Our study extends these findings by incorporating two additional dimensions: circadian rhythm association and immune microenvironment modulation. Furthermore, this work systematically explores the expression profiles of circadian rhythm-related genes in COPD and their correlation with immune microenvironment features. We also constructed a preliminary ceRNA regulatory network for the characteristic genes and predicted potential therapeutic targets. These findings provide novel insights and a robust data foundation for elucidating the molecular mechanisms underlying COPD and for developing targeted intervention strategies.

Functional enrichment analysis (GO/KEGG) revealed that DECRRGs not only participate in circadian clock regulation but also critically intervene in the core pathophysiological processes of COPD by integrating the “rhythm-immune-metabolism” axis. These genes were significantly enriched in key pathways governing central COPD pathologies, including the IL-1 signaling pathway mediating chronic inflammation (a key driver of COPD pathology [28]), pathways associated with neuromuscular transmission and skeletal muscle dysfunction (e.g., respiratory muscle weakness and reduced exercise tolerance [29]), and pathways involving coagulation abnormalities associated with prothrombotic states during acute exacerbations and immune regulation of the coagulation system [30,31]. Additionally, DECRRGs are significantly enriched in the C-type lectin receptor (CLR) signaling pathway, a core pathway for innate immune recognition and inflammatory regulation [32,33]. Collectively, circadian rhythm dysregulation is associated with immune‑inflammatory activation and metabolic imbalance, particularly impaired energy metabolism.

In the validation cohort, CHRNA1 demonstrated consistent and significant upregulation across cohorts, highlighting its robustness and research value as a core circadian-related gene. Furthermore, single‑cell transcriptomic analysis predominantly localized CHRNA1 to B cells, a pattern observed in both COPD patients and healthy controls.

CHRNA1 encodes the α1 subunit of the nicotinic acetylcholine receptor (nAChR), a key component of acetylcholine (ACh) signaling at the neuromuscular junction that is essential for maintaining normal neuromuscular function [34,35]. This study explored the potential associations of aberrant CHRNA1 expression with muscle complications in COPD (such as sarcopenia [29]), and alterations in the pulmonary immune microenvironment. It has been reported that CHRNA1, through nicotinic acetylcholine receptors (nAChRs), participates in regulating airway smooth muscle tone, mucus secretion, and inflammatory responses [36], and may influence skeletal muscle mitochondrial function and energy metabolism [37,38]. The findings of this study suggest that CHRNA1, as a gene integrating circadian regulation and neuromuscular function, may link circadian disruption, muscle dysfunction, and alterations in the pulmonary immune microenvironment in COPD.

The diagnostic model constructed based on the eight feature genes (including CHRNA1) demonstrated moderate discriminatory ability (AUC = 0.856, 95% CI: 0.806–0.902), suggesting its potential utility in risk stratification. Owing to its high feature importance and stable expression across datasets, CHRNA1 showed potential value as a biomarker. As an exploratory computational analysis, drug prediction using the DSigDB database identified compounds that may target these feature genes, including amitriptyline and fluoxetine (whose cognitive enhancement potential requires further investigation in COPD patients with prevalent cognitive impairment [39]), as well as rocuronium (for which the risk of respiratory depression due to its neuromuscular blocking effects requires careful evaluation [26]). These findings should serve as preliminary hypotheses for future research rather than clinical conclusions. Furthermore, the constructed mRNA–miRNA–lncRNA (ceRNA) regulatory network, integrating the critical role of non‑coding RNAs in circadian regulation [40,41] and advances in emerging RNA therapeutics [42], provides a theoretical foundation and potential targets for deepening the understanding of COPD pathomechanisms—particularly the “circadian‑immune‑metabolic” axis and CHRNA1‑related pathways—as well as for the development of non‑coding RNA‑based targeted intervention strategies.

It should be noted that the circadian rhythm gene set used in this study was obtained from MSigDB, and some genes within this set (e.g., IL6) are classically associated with immune‑inflammatory regulation. Therefore, the enrichment of differentially expressed circadian rhythm genes in immune pathways may be partly influenced by the intrinsic composition of the gene set itself.

To avoid circular reasoning, we did not over‑rely on pathway enrichment analysis of the full gene set. Instead, we focused on CHRNA1, which is classically involved in neuromuscular junction signaling rather than being a conventional immune gene. The association between CHRNA1 and the COPD immune microenvironment was further validated through multiple independent approaches, including independent cohort validation, single‑cell localization, and immune infiltration correlation analyses. Therefore, the conclusion that CHRNA1 may link circadian rhythms and immune regulation is not driven solely by gene set definitions.

### 4.1. Limitations and future directions

This study has several limitations. First, our analysis relied on publicly available microarray data, which carries risks of batch effects and limited sample sizes. Although we performed batch correction and addressed the non-independence issue in GSE151052 by using it only for validation, independent prospective cohorts are still needed. Second, while CHRNA1 was identified from an MSigDB circadian gene set, direct experimental evidence of its 24-hour rhythmic expression in COPD is lacking and requires future time-series experiments. Third, our immune infiltration analysis used CIBERSORT on bulk transcriptomic data, which provides estimated rather than direct quantification. Validation by flow cytometry or immunohistochemistry in independent cohorts is warranted. Fourth, given the systemic nature of COPD, our analysis focused primarily on lung tissue. Importantly, all findings are correlational rather than causal.

Future work should integrate in vitro and in vivo experiments with clinical studies to establish causal mechanisms. Prospective cohorts are needed to validate the diagnostic model; flow cytometry or immunohistochemistry to confirm the immunological findings; and functional assays (e.g., CHRNA1 knockdown/overexpression) to establish causality. Additionally, future studies should include multiple tissue types (e.g., peripheral blood, skeletal muscle) to better understand the systemic implications of circadian disruption in COPD.

## 5. Conclusion

This study suggests that circadian rhythm‑related genes, particularly CHRNA1, may participate in the pathogenesis of COPD through neuro‑immune crosstalk. The exploratory risk stratification model constructed based on the eight feature genes demonstrated favorable risk stratification capability. Importantly, this model represents a hypothesis-generating tool for COPD risk assessment rather than a clinically validated diagnostic instrument; prospective validation in independent cohorts incorporating clinical parameters is required before any clinical application. The established ceRNA regulatory network provides a theoretical framework for further understanding the molecular mechanisms underlying the “circadian‑immune‑metabolic” axis in COPD. Future studies should combine organoid models and prospective COPD circadian rhythm cohorts to systematically validate the temporal expression dynamics of CHRNA1 and its interactions with key immune cell subsets (e.g., B cells, M1 macrophages, CD8 ⁺ T cells), as well as to elucidate the causal regulatory mechanisms involved.

## Supporting information

S1 Table List of circadian rhythm-related genes. Nine gene sets were retrieved from MSigDB (Gene Set IDs: M22067, M14104, M13729, M12080, M18009, M95, M938, M39605, M36019). After removing duplicate genes, these sets were consolidated into a reference list of 1,010 unique genes.
(XLSX)

S1 File Code. (ZIP)
